# Brain-Derived Neurotrophic Factor in Patients with Huntington's Disease

**DOI:** 10.1371/journal.pone.0022966

**Published:** 2011-08-12

**Authors:** Chiara Zuccato, Manuela Marullo, Barbara Vitali, Alessia Tarditi, Caterina Mariotti, Marta Valenza, Nayana Lahiri, Edward J. Wild, Jenny Sassone, Andrea Ciammola, Anne Catherine Bachoud-Lèvi, Sarah J. Tabrizi, Stefano Di Donato, Elena Cattaneo

**Affiliations:** 1 Department of Pharmacological Sciences and Center for Stem Cell Research, Università degli Studi di Milano, Milan, Italy; 2 Division of Biochemistry and Genetics, National Neurological Institute-IRCCS “Carlo Besta”, Milan, Italy; 3 Department of Neurodegenerative Disease, UCL Institute of Neurology, University College London and National Hospital for Neurology and Neurosurgery, London, United Kingdom; 4 IRCCS Istituto Auxologico Italiano, Milano, Italy; 5 AP-HP, Centre National de référence maladie de Huntington, GHU Chenevier Mondor, Créteil, France; Emory University, United States of America

## Abstract

Reduced Brain-Derived Neurotrophic Factor (BDNF) levels have been described in a number of patho-physiological conditions, most notably, in Huntington's disease (HD), a progressive neurodegenerative disorder. Since BDNF is also produced in blood, we have undertaken the measurement of its peripheral levels in the attempt to identify a possible link with HD prognosis and/or its progression. Here we evaluated BDNF level in 398 blood samples including 138 controls, 56 preHD, and 204 HD subjects. We found that BDNF protein levels were not reliably different between groups, whether measured in plasma (52 controls, 26 preHD, 105 HD) or serum (39 controls, 5 preHD, 29 HD). Our experience, and a re-analysis of the literature highlighted that intra-group variability and methodological aspects affect this measurement, especially in serum. We also assessed BDNF mRNA levels in blood samples from 47 controls, 25 preHD, and 70 HD subjects, and found no differences among the groups. We concluded that levels of BDNF in human blood were not informative (mRNA levels or plasma protein level) nor reliable (serum protein levels) as HD biomarkers. We also wish to warn the scientific community in interpreting the significance of changes measured in BDNF protein levels in serum from patients suffering from different conditions.

## Introduction

Reductions in Brain-Derived Neurotrophic Factor (BDNF) levels have been implicated in various pathological conditions affecting different organs [1], [2], [3], [4], [5]. BDNF has been detected also in the blood [6], [7], [8]. Because of its accessibility, interest has been kindled in the assessment of BDNF protein in blood, with the primary goal of determining whether its levels correlate to a given pathology and/or its progression or response to drugs. A summary of the most critical papers reporting such measurement is presented in **Table S1** (BDNF measurements in human serum) **and S2** (BDNF measurement in human plasma). In particular, low brain BDNF levels have been described in various neurodegenerative disorders, most notably, Huntington's Disease (HD), a progressive neurodegenerative disorder caused by a CAG trinucleotide expansion in the gene that encodes huntingtin [9], [10].

Reduced level of BDNF protein and mRNA have been found in HD cells, in brain tissues from HD mice and in human post-mortem material [10]. A similar reduction in BDNF mRNA level was found in HD rodent blood [11]. Furthermore, treatment of HD mice with the MLK inhibitor CEP-1347 led to increase in total BDNF mRNA in the blood and brain [11], [12]. Recently, some of the current authors (JS, AC) have reported that serum BDNF was significantly lower in patients with HD than in age-matched healthy subjects [13] and other authors reported significantly higher levels in patients treated with riluzole [14]. However, intra-group variability may be problematic, especially since BDNF protein level is sensitive to a variety of factors [15], [16], [17], [18]. Here we have undertaken a study aimed at evaluating BDNF level in the peripheral blood of HD patients, in order to investigate its robustness as a biological predictor of HD prognosis and drug efficacy.

## Materials and Methods

### Study design

Subjects were recruited from the National Neurological Institute “Carlo Besta”, Milan (Italy), the National Hospital for Neurology and Neurosurgery (London UK), the IRCCS Istituto Auxologico Italiano, Milan (Italy), and the Henri Mondor Hospital, Créteil (France). In total, samples from three Italian Cohorts (A,B for serum measurements; C for whole blood detection), one French Cohort (for detection in plasma) and three UK Cohorts (A, for plasma detection; B,C for whole blood) were collected and analyzed (see **Table 1** and **2** ).

**Table 1:** Clinical and genetic characteristics of HD, preHD and healthy patients.

Samples			N°	Gender (F/M)	Age	CAG Size
**Serum**	**Italian Cohort A**	controls	32	20/12	43(28–69)	-
		preHD	5	3/2	-	42(38–44)
		HD	22	8/14	53(32–79)	45(39–51)
	**Italian Cohort B**	controls	7	-	39(36–58)	-
		HD	7	-	53(35–75)	43(41–45)
**Plasma**	**French Cohort**	controls	22	-	55(26–82)	-
		preHD	4	2/2	39(36–41)	43(42–44)
		HD*	40	21/15	44(25–65)	44(40–50)
	**UK Cohort A**	controls	30	19/11	47(26–72)	-
		preHD	22	11/11	38(23–53)	43(40–47)
		HD	65	35/30	48(22–80)	45(39–64)
**Whole blood**	**UK Cohort B**	controls	8	4/4	45(28–63)	-
		preHD	10	7/3	42(27–54)	42(40–44)
		earlyHD	11	5/6	48(33–67)	43(40–48)
		moderateHD	11	7/4	50(28–63)	49(41–56)
	**UK Cohort C**	controls	16	7/9	46(25–68)	-
		preHD	11	5/6	44(28–56)	42(38–44)
		earlyHD	15	10/5	49(33–65)	43(40–49)
		moderateHD	9	6/3	65(29–77)	48(41–56)
	**Italian Cohort C**	controls	23	13/10	36(21–60)	-
		preHD	4	3/1	36(28–42)	46(40–47)
		HD	24	12/12	47(28–68)	45(41–83)

Italian Cohort A, Istituto Auxologico Italiano, Milano; Italian Cohort B, National Neurological Institute “Carlo Besta” Milan; French Cohort, Henry Mondor Hospital, Creteil; UK Cohort, National Hospital for Neurological and Neurosurgery.

*sex, age and CAG missing for 4 samples. Age and CAG size are expressed as median values (min-max).

**Table 2:** Medical treatments.

Samples			NLPs	BDZs	SSRIs	SSNRIs	TCAs	Other	No drugs
**Serum**	Italian Cohort A	HD	6	4	5	1	-	7	4
		preHD	-	-	-	-	-	-	5
	Italian Cohort B	HD	3	2	3	-	-	2	4
		preHD	-	-	-	-	-	-	-
**Plasma**	French Cohort	HD	10	6	9	-	5	11	16
		preHD	-	-	-	-	-	-	4
	UK Cohort A	HD	7	1	7	1	-	10	7
		preHD	-	-	-	-	-	-	-
**Blood**	UK Cohort B	HD	8	4	4	1	2	13	3
		preHD	-	-	2	-	-	1	7
	UK Cohort C	HD	3	-	8	1	-	5	8
		preHD	-	-	3	-	-	2	7
	Italian Cohort C	HD	15	7	8	3	1	4	11
		preHD	-	-	-	-	-	-	4

NLPs, neuroleptics; BDZ, benzodiazepines; SSRIs, selective serotonin reuptake inhibitors; SSNRIs, selective serotonin-norepinephrine reuptake inhibitors; TCAs, tricyclic antidepressants.

### Ethics statement

All eligible participants received verbal and written information about the study, and provided signed, informed consent, according to the Declaration of Helsinki. Department of Neurodegenerative Disease, UCL Institute of Neurology University College London and National Hospital for Neurology and Neurosurgery, London: the study was approved by National Hospital of Neurology and Neurosurgery & Institute of Neurology Joint Research Ethics Committee (Central London REC 3). Centre National de référence maladie de Huntington, GHU Chenevier Mondor, Créteil: the study was approved by the CPP (Comité de protection des personnes) Henri Mondor, Créteil, France. National Neurological Institute-IRCCS “Carlo Besta”: the study was approved by the Istituto Neurologico “Carlo Besta” Institutional Review Board. IRCCS Istituto Auxologico Italiano, Milano: the study was approved by The Ethics Committees of IRCCS Istituto Auxologico Italiano “Comitato Etico dell'IRCCS Istituto Auxologico Italiano di Milano”.

### Patients exclusion/inclusion criteria

#### Symptomatic HD patients

This group included patients that were positive on the molecular test for the presence of a CAG triplet with >35 repeats in the Huntington gene. They also manifested clinical signs and symptoms of HD. Clinical assessments of motor signs, disease staging and total functional capacity (TFC) were determined with the Unified Huntington's Disease Rating Scale (UHDRS). The age at onset was considered the time when clear motor signs and symptoms were first noticed.

#### Pre-HD individuals

We enrolled subjects that had more than 35 CAG triplet repeats in the Huntington gene, with no clinical symptoms or signs of the disease, and a UHDRS motor diagnostic confidence score of <4. For each pre-HD individual, the estimated probability of onset within the next 5 years was calculated on the basis of the number of CAG repeats and his/her present age, according to Langbehn [19].

#### Control individuals

This group included volunteers with no neurodegenerative disorders. Patients and controls were excluded from the study who were <18 years old and had known major medical conditions in addition to the primary genetic disorder.

### Blood sampling and processing

For patients and control subjects from Henri Mondor Hospital, Créteil, and from the Neurological Institute-IRCCS “Carlo Besta” in Milan, blood samples were always withdrawn after an overnight fast. For patients from IRCCS Istituto Auxologico Italiano in Milan, blood samples were withdrawn after breakfast. From UCL Institute of Neurology, London, non-fasted samples were withdrawn.

### Plasma

French Cohort. 5 ml of blood were collected in EDTA-treated tubes between 9:00 and 12:00 a.m. Blood samples were maintained at room temperature (RT; 20–25°C) until plasma separation, which was performed up to 4 to 5 hours later. Tubes were centrifuged for 20 minutes at 3000×g in order to separate plasma from cellular components; plasma samples were stored in 1 ml aliquots at −80°C. Control, HD, and preHD subjects were recruited on the same day.

UK Cohort A. 5 ml of blood were collected in EDTA-treated tubes between 2:00 pm and 5:00 pm. Blood samples were maintained at RT until plasma separation, which was performed within 2 hours. Blood was transferred to a histopaque tube (A6929 ACCUSPIN™ System-HISTOPAQUE®-1077 Sigma Aldrich, St. Louis, MO, USA) and centrifuged for 10 minutes at 1000×g in order to separate plasma from cellular components; plasma samples were stored in 1 ml aliquots at −80°C and shipped in 200 µl aliquots.

### Serum

Italian Cohort A. 3 ml of blood were collected between 9:00 and 12:00 a.m., after breakfast. Blood samples were maintained at RT for 1 hour, and serum was separated by centrifugation at RT for 10 minutes (2000×g). Serum samples were stored in 150 µl aliquots at −80°C.

Italian Cohort B. 3 ml of blood were collected between 9:00 and 12:00 a.m., after an overnight fast. Blood samples were maintained at RT for 1 hour, and serum was separated by centrifugation at RT for 10 minutes (2000×g). Serum samples were stored in 150 µl aliquots at −80°C.

### RNA

UK Cohort B, C and Italian Cohort C. Whole venous blood was collected in an evacuated blood collection tube (PAXgene™ Blood RNA Tube, BD, Franklin Lakes, NJ, USA) that contained a stabilizing additive between 2:00 pm and 5:00 pm. The PAX tubes were left at room temperature for 2 hours as per Qiagen protocol and then stored at −80°C until RNA extraction.

### BDNF ELISA

Plasma and serum samples were assayed for BDNF protein levels with the BDNF Emax ImmunoAssay System (Promega, Madison WI, USA). In order to minimize inter-sample variance resulting from experimental variation, all samples were measured simultaneously and with BDNF ELISA kits from the same batch. Internal standards for the BDNF ELISA kit were used as suggested by [20] and consisted of (a) a standard curve performed in each experiment to allow BDNF protein quantification, and (b) one standard sample present on all plates. Plasma and serum aliquots were thawed on the same day of the experiment at 9:00 a.m. Plasma was diluted at 1∶20 in PBS1X; serum samples were diluted at 1∶300 in PBS1X. Each plasma and serum sample was tested for BDNF content in three independent ELISA assays. Plasma and serum dilutions were tested in triplicate on each ELISA plate. A Synergy HT Multi-Detection Microplate Reader was used to read absorbance at 450 nm.

### RNA extraction

Total RNA was extracted from whole blood with the PAXgene™ Blood RNA System Kit (PreAnalytiX, Hombrechtikon, CH). Total RNA from brain was isolated from 200–300 mg of human brain tissue with 2 ml of TRIZOL reagent (Invitrogen, Carlsbad, CA, USA). The tissue was then homogenised in liquid nitrogen with a mortar and pestle. To enhance the RNA yield, the samples were precipitated by adding 2 µl of glycogen solution (10 mg/ml) in isopropanol, and incubating at −80°C overnight. Total RNA concentration was measured with a NanoDrop 1000 Spectrophotometer (Thermo Scientific, Waltham, MA, USA). The RNA quality was verified by electrophoresis of 1 µg from each sample on an agarose gel. The total RNA was stored in aliquots at −80°C.

### Reverse transcription of RNA

250 ng of total RNA was reverse-transcribed to single-stranded cDNA with Superscript III RNaseH- reverse transcriptase (Invitrogen, Carlsbad, CA, USA) and random primers in a volume of 20 µl, according to the manufacturer's instructions. Two independent reverse transcription reactions were performed for every RNA stock. For the analyses of BDNF mRNA isoforms in whole blood, 2 µg of total RNA was reverse-transcribed to single stranded cDNA. For the analyses of BDNF mRNA in the cortex, 1 µg of total RNA was reverse-transcribed to single stranded cDNA.

### Real-time PCR

Three independent PCR analyses were performed for each of two independent reverse transcription reactions; thus, a total of six independent measurements were performed for each of the analysed mRNA. We used an iCycler Thermal Cycler with a Multicolour Real-time PCR Detection System (Bio-Rad, Hercules, CA, USA). All reactions were performed in a total volume of 25 µl that contained 5 µl of cDNA (1∶10 dilution of reverse transcribed samples for BDNF, ROCK1, ANXA1, GAPDH, B2M and ß-actin amplification or 1∶200 dilution of reverse transcribed samples for EAR amplification), 50 mM KCl, 20 mM Tris-HCI, pH 8.4, 0.2 mM dNTPs, 25 units/ml iTaq DNA polymerase, 3 mM MgCl_2_, SYBR Green I with 10 nM fluorescein and stabilisers (iQTM SYBR Green Supermix-Biorad, Hercules, CA, USA), and 0.3 µM each of forward and reverse primers. The amplification cycles consisted of an initial denaturing cycle at 95°C for 3 minutes, followed by 45 cycles of 30 s at 95°C, 30 s at 60°C, and 30 s at 72°C. Fluorescence was quantified during the 60°C annealing step, and product formation was confirmed with a melting curve analysis (55°C–94°C).

The BDNF-specific primers were based on the BDNF coding sequence (GenBank accession number AF411339), as follows: BDNF 5′-TAACGGCGGCAGACAAAAAGA-3′; BDNF 5′-GAAGTATTGCTTCAGTTGGCCT-3′. The amplification product was 101 base pairs (bp) long. The expressed Alu repeat (EAR)-specific primers were based on the human interspersed Alu repetitive sequence. The primer sequences were: EAR 5′-GAGGCTGAGGCAGGAGAATCG-3′ EAR 5′-GTCGCCCAGGCTGGAGTG-3′. The amplification product was 87 bp long. The ANXA1 specific primers were set up referring to GenBank accession number NM_000700.1. The primers sequences were Fw: 5′- GAGCCCCTATCCTACCTTCAAT-3′; Rev: 5′-GATGGTTGCTTCATCCACACCT-3′. The amplification product was 88 bp long. The ROCK1 specific primers were set up referring to GenBank accession number NM_005406.2.

The primers sequences were Fw: 5′-AGGTTAGGGCGAAATGGTGTAGAA-3′; Rev: 5′-CCTCTCCTTTATCTTCTTCCAAGTC-3′. The GAPDH specific primers were set up referring to GenBank accession number NC_000012.10. The primer sequences were: GAPDH Fw: 5′-AGCTGAACGGGAAGCTCACT-3′ GAPDH Rev: 5′-AGGTCCACCACTGACACGTTG-3′. The amplification product was 67 bp long. The B2M specific primers were set up referring to GenBank accession number NC_000015.8. The primer sequences were: B2M Fw: 5′-GGAGAGAGAATTGAAAAAGTGGAGC-3′; B2M Rev: 5′-GGCTGTGACAAAGTCACATGGTT-3′. The amplification product was 143 bp long. The ß-actin specific primers were set up referring to GenBank accession number NC_000007.12. The primer sequences were: Fw: 5′- AGTGTGACGTGGACATCCGCA-3′; Rev: 5′- GCCAGGGCAGTGATCTCCTTCT-3′. The amplification product was 112 bp long.

### Qualitative PCR

Semi-quantitative PCRs were performed in a total volume of 50 µl, consisting of 100 ng of cortex cDNA or 200 ng of blood cDNA, 20 mM Tris-HCl pH 8.4, 50 mM KCl, 1.5 mM MgCl_2_, 0.2 mM dNTPs, 0.4 µM each of forward and reverse primers, and 2.5 units of Taq polymerase (Invitrogen). The amplification cycles consisted of an initial denaturing cycle at 94°C for 5 minutes, followed by 35 cycles (or 40) of 1 minute at 94°C, 45 s at different temperatures, according to the melting temperature of the primers, and 45 s at 72°C. The sequences of all primers, their annealing temperatures, and the lengths of the PCR products are provided upon request.

### Statistical analyses

As BDNF values do not have a normal distribution in both control and HD populations, the non-parametric Kruskal-Wallis test followed by Dunn's Multiple comparison test or Mann-Whitney two- tailed U-test were used for the statistical analyses, with significance being set at P<0.05. Median values were chosen because of the skewed distribution of the data. In the box plots, the boundary of the box closest to zero indicates the 25th percentile, the line within the box marks the median, and the boundary of the box farthest from zero indicates the 75th percentile. When 10 or more samples were analyzed, whiskers appear above and below the box to indicate the 90th and 10th percentiles. Unpaired t-test and Spearman's rank correlation coefficient were also used.

## Results

### BDNF protein detection

#### BDNF in plasma

**Figure 1:**
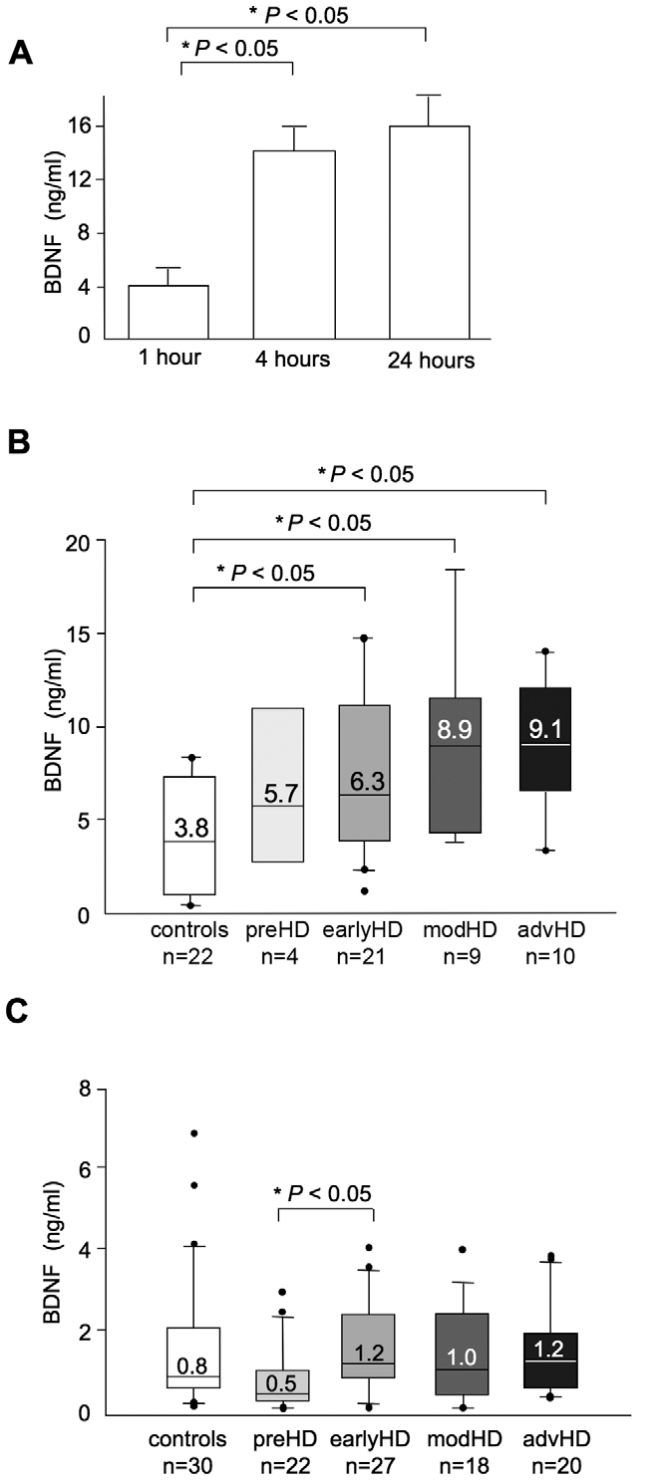
BDNF protein in human plasma measured by ELISA. (**A**) BDNF content in plasma samples from 3 control subjects. After blood withdrawal, samples remained at RT for 1, 4, and 24 hours before the plasma was isolated (p<0.05 compared to sample processed after 1 hour, unpaired t-test). (**B**) BDNF protein levels in plasma samples from the French Cohort, prepared 4 hours after blood withdrawal. Control and HD subjects exhibited a large variability in plasma BDNF levels. Values inside the boxes are the medians for the group. P<0.05, controls vs early HD, P<0.05 controls vs moderate HD and P<0.05 controls vs advanced HD, according to Kruskal-Wallis test followed by Dunn's multiple comparison test (**C**) BDNF protein contents in plasma samples from UK Cohort A, prepared 2 hours after blood withdrawal. BDNF protein in preHD was significantly lower than that in early HD, P<0.05 according to Kruskal-Wallis test followed by Dunn's multiple comparison test.

In a preliminary ELISA assay we noticed that the time between withdrawal and plasma preparation affected the detectable BDNF levels in plasma. Plasma prepared 1 hour after blood withdrawal showed BDNF values of 4±2.4 ng/ml that were lower than those prepared at 4 hours (14±2.1 ng/ml) and 24 hours (16±2.4 ng/ml) after withdrawal (**Fig. 1A**) (P<0.05 compared to samples processed after 1 hour, unpaired t-test). The higher than expected BDNF levels measured in these plasma samples could be due to BDNF release from platelets [7].

To devise a more standardised plasma preparation, and to ensure a significant number of samples available for testing, we conducted our first study on plasma samples from the Blood Bank of the Henry Mondor Hospital in Creteil (French Cohort, see **Table 1**). The subjects included 40 with HD, 4 with preHD, and 22 controls. As shown in **Fig. 1B**, an increase in plasma BDNF protein level was observed in HD patients at early, moderate and advanced HD stages, compared to controls (P<0.05 by Kruskal-Wallis test followed by Dunn's multiple comparison test **Fig. 1B**). Although the median value of BDNF content tended to increase from the early to moderate or advanced stages, the difference between these groups was not significant (P>0.05 by Kruskal-Wallis test followed by Dunn's multiple comparison test). Furthermore, we found no correlations between BDNF content and disease progression measured by TFC and UHDRS scores, or the CAG-repeat length (P>0.05, Spearman test). Antidepressants were reported to increase BDNF levels in the serum of depressed subjects [21]. We found no differences in BDNF content between antidepressant-free and antidepressant-treated HD subjects (P>0.05, Mann-Whitney two-tailed U-test).

To evaluate further whether increased levels of blood BDNF protein were consistently detected in human HD blood, we increased the number of subjects tested. A new batch of plasma samples was obtained from University College London (UCL, UK Cohort A, **Table 1**), including 30 controls, 22 preHD, and 65 HD. According to UCL internal procedures, plasma preparations for the UK Cohort A were performed differently than those prepared for the French Cohort. Blood samples were collected after breakfast (and not after an overnight fast as for the French Cohort) and plasma samples were prepared within 2 hours of blood withdrawal (compared to 4 hours after for the French Cohort). **Fig. 1C** shows that the median BDNF level in plasma from control subjects of the UK Cohort A was 0.8 ng/ml compared to the 3.8 ng/ml measured in plasma samples from controls of the French Cohort (**Fig. 1B**). This is likely to be due to differences in samples preparation. The lower than expected BDNF levels found in the UK cohort could be due to lower platelet activation and minimal BDNF release. Therefore, we analysed the samples from these two cohorts separately. We found no difference in BDNF levels between the HD and control populations (P>0.05 by Kruskal-Wallis test followed by Dunn's multiple comparison test; **Fig. 1C**). This contrasted with the results from the French Cohort, which indicated that the BDNF protein content was significantly higher in HD patients compared to controls (**Fig. 1B**). The most likely explanation for these different results was the different protocols used in the plasma preparation and the many variables related to the patients recruitment. Further, we found that BDNF protein content in the UK Cohort A did not correlate with disease progression. Additionally, we found no difference in BDNF content between antidepressant-free and anti-depressant treated HD subjects (P>0.05, Mann-Whitney two-tailed U-test). Our analyses revealed also that BDNF protein in 22 preHD subjects was lower than in early HD (P<0.05 by Kruskal-Wallis test followed by Dunn's multiple comparison test; **Fig. 1C**). We calculated the 95% probability that preHD subjects would exhibit onset symptoms within 5 years (based on the number of CAG repeats) [19]. We found a tendency of BDNF to decrease in subjects predicted to be near disease onset, but the correlation was not significant (P>0.05, Spearman test). Five out of the 8 subjects close to onset (5-year probability >0.3) had BDNF levels in the lower range while the other 3 subjects, which exhibited BDNF levels higher or similar to controls, were predicted to be further away from onset (**Figure S1**).

In conclusion, control and HD subjects from two independent Cohorts showed completely different BDNF levels. This is likely to be due to differences in samples preparations or intra-group variability, because BDNF levels are sensitive to a variety of factors (for example age and sex) [15], [16], [17], [22]. However, no sex- and age-related changes in BDNF blood concentration were found both in control and HD populations.

#### BDNF in serum

**Figure 2:**
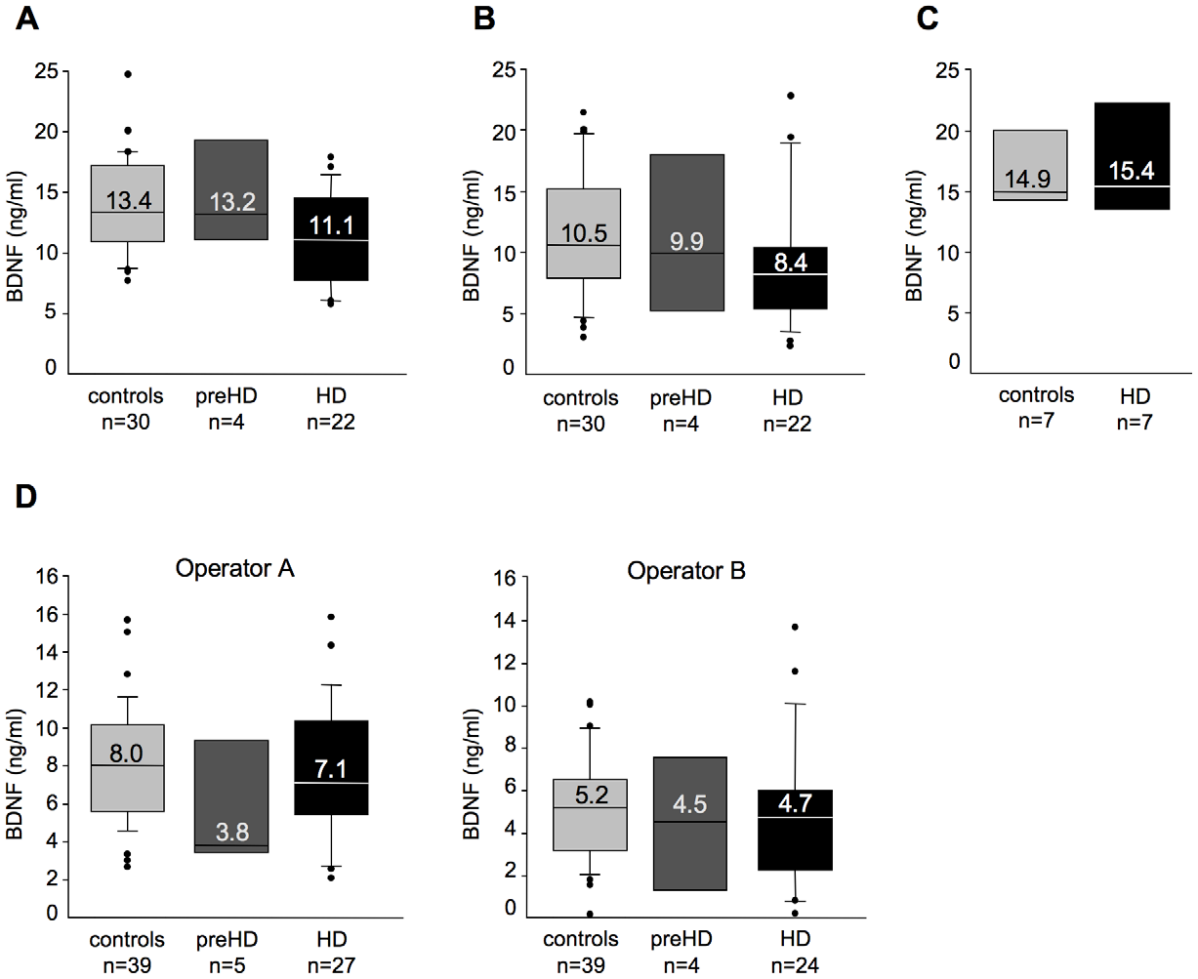
BDNF protein measured in human serum. (**A**) BDNF protein in serum from controls and subjects with preHD or HD in Italian Cohort A. (**B**) The samples in A were tested 1 week later. (**C**) BDNF levels in control and HD serum samples from the Italian Cohort B. (**D**) Serum BDNF content determined 3 months later. The following samples were tested: (i) thirty-nine controls, including 32 from Italian Cohort A and 7 from Italian Cohort B. From Italian Cohort A 30 had been tested previously and 2 were new samples. All those from Italian Cohort B had been tested previously; (ii) five with preHD from Italian Cohort A, including 4 samples that were previously tested and one new sample; (iii) twenty-seven with HD, including 20 out of 22 samples from Italian Cohort A and 7 samples from Italian Cohort B. Duplicate experiments were performed in parallel by two different operators (operator A, JS; operator B, BV). Operator B: BDNF protein content was undetectable in 1 preHD sample and in 3 HD samples which were excluded from data analyses.

A previous report from some of the authors (AC and JS) of this paper indicated a decrease in BDNF level in human HD blood [13]. We have asked this group to join our study and to provide us with their old/new samples (control and HD) and/or re-perform this measurement. A first cohort (Italian Cohort A, see **Table 1**) including 32 controls, 5 preHD, and 22 HD patients was recruited by AC Unit at Istituto Auxologico in Milan and BDNF content was first evaluated in July 2009 in a subgroup including 29 control and 19 HD samples. A not significant decreasing tendency in serum BDNF content was observed in HD subjects compared to controls (median BDNF level in serum from controls was 10.7 ng/ml compared to 5.9 ng/ml in HD subjects; P>0.05 compared to controls, Mann-Whitney Test). In October 2009 a second evaluation on the same samples (new aliquots of serum), added of 1 control, 4 preHD and 3 HD, was performed by other authors on this paper (BV and CZ). Again, serum BDNF level was similar in control and HD patients (P>0.05, Kruskal-Wallis test followed by Dunn's multiple comparison test) (**Fig. 2A**). Because the variability in serum BDNF content was high in both the control and HD populations, a replicate of this experiment was performed 6 days later with the same ELISA kit and reagents. This experiment confirmed the high variability in serum BDNF in both groups, and the absence of a significant difference in serum BDNF levels between control and HD patients (P>0.05) according to Kruskal-Wallis test followed by Dunn's multiple comparison test)(**Fig. 2B**). We also observed that 16 out of 30 controls and 20 out of 22 patients with HD showed significant differences between the first and second measurements (>15%). These results suggested that caution should be taken when interpreting data on BDNF serum, because inter-assay variation is very high despite our best efforts at methodological consistency (**Text S1**). In parallel, we tested a small group of serum samples (7 from controls and 7 from HD patients, Italian Cohort B, see **Table 1**) obtained by CM and SD at the National Neurological Institute “Carlo Besta” in Milan. Again, no differences in the BDNF content were observed between control and HD subjects (P>0.05, Mann-Whitney Test) (**Fig. 2C**).

To try to limit inter-assay variations in the BDNF analyses on serum we have organized our assay in order to test all serum samples from Italian Cohorts A and B (new serum aliquots that were never thawed before) on the same day and BV and JS have worked in parallel with the same sample dilutions, ELISA assays, reagents, solutions, and bench. This experiment was performed in January 2010. As shown in **Fig. 2D**, both operators found no significant differences in serum BDNF protein content between control, pre-HD and HD subjects (JS: P>0.05; BV: P>0.05, Kruskal-Wallis test followed by Dunn's multiple comparison test). Inter-assay variation still remained very high (**Text S1**). Moreover, the absolute amounts of BDNF content in serum were lower in this measurement than those found in the previous measurement (**Fig. 2A**), suggesting that the storage conditions (−80°C) affected BDNF stability in serum samples. These data suggested that measurement of BDNF protein in human serum should be interpreted with extreme caution. Further, in contrast to previous findings [13], we did not detected any difference in serum BDNF content between control and HD patients.

### BDNF mRNA in control and HD patients

**Figure 3:**
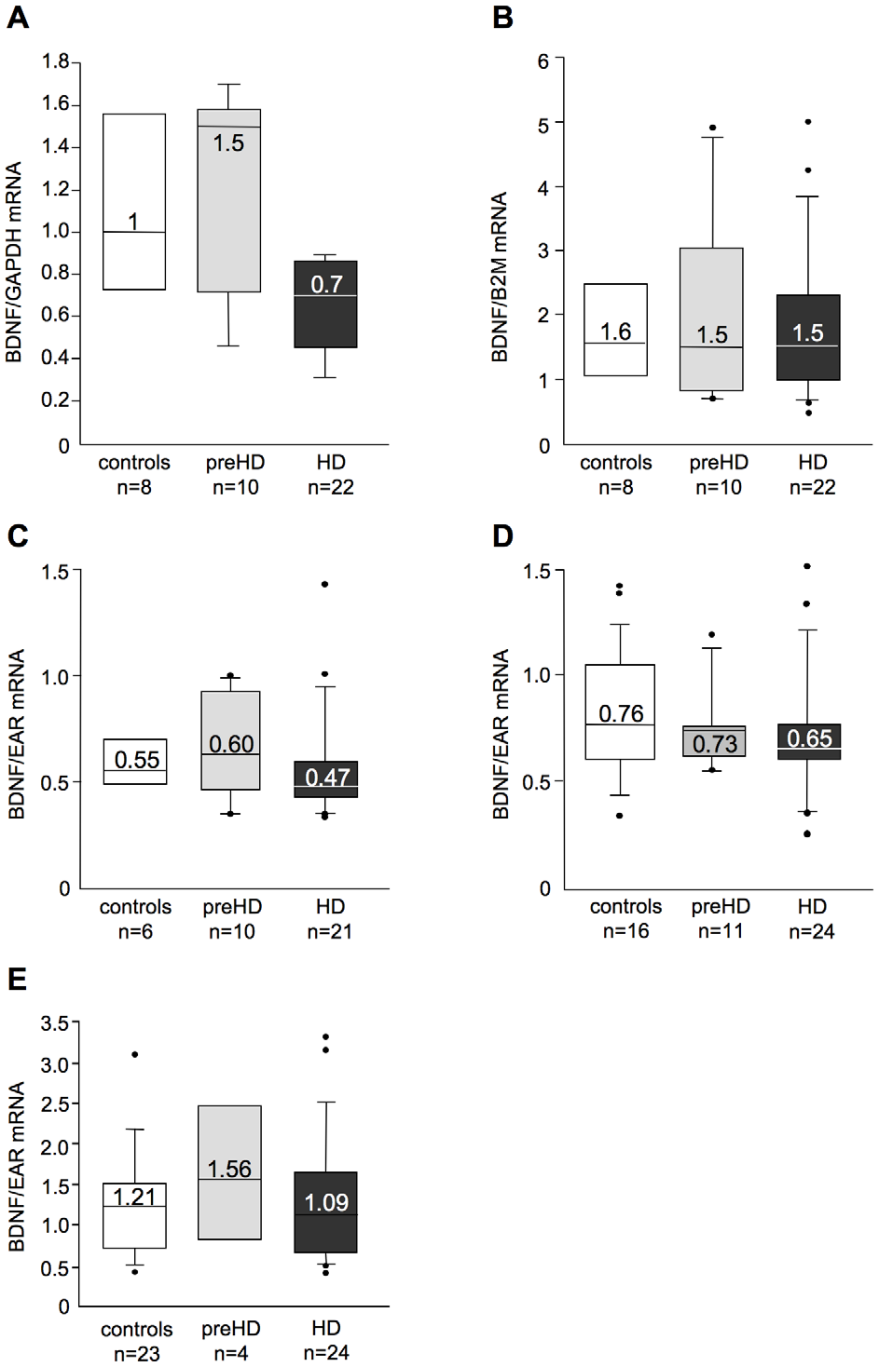
Analyses of BDNF mRNA in whole blood from control and HD subjects. (**A**) Samples from UK Cohort B: BDNF mRNA levels normalised with GAPDH mRNA. (**B**) Samples from UK Cohort B: BDNF mRNA levels normalised with ß2-microglobulin (B2M) mRNA. (**C**) BDNF mRNA content in the same samples shown in (A) was normalised with EAR. (**D**) Samples from UK Cohort C: BDNF mRNA was normalised with EAR. (**E**) Samples from Italian Cohort C: BDNF mRNA was normalised with EAR.

There is a remarkable reduction in brain BDNF mRNA levels in patients with HD as judged from analyses of post-mortem tissue [23]. Studies in mouse models of HD indicated that blood BDNF mRNA also gradually diminishes with disease progression [11], [12]. Due to our failure to detect reliably the BDNF protein in human blood, we decided to measure BDNF mRNA in the blood of patients with HD and controls. In our initial RT-qPCR assays, we measured BDNF mRNA levels in the blood of 8 controls, 10 preHD, and 22 HD subjects (UK Cohort B, see **Table 1**). BDNF mRNA levels showed a tendency to decrease in HD patients with respect to controls and preHD samples after normalisation to the mRNA of the housekeeping gene, glyceraldehyde-3-phosphate dehydrogenase (GAPDH), although this difference was not statistically significant (P>0.05 by Kruskal-Wallis test followed by Dunn's multiple comparison test; **Fig. 3A**). Similarly, when BDNF mRNA from the same samples was normalised to ß2-microglobulin, a component of major histocompatability class I molecules present in all nucleated cells, we found that BDNF mRNA levels were not significantly different among control, preHD, and HD (P>0.05 by Kruskal-Wallis test followed by Dunn's multiple comparison test) (**Fig. 3B**). Since we have previously demonstrated the possibility of erroneous quantification of a target gene in human blood when a conventional normalization strategy based on a single control gene is used [24], we decided to re-analyse the samples still available by using a more accurate strategy for mRNA normalisation in RT-qPCR based on amplification of the expressed Alu repeat (EAR) [24]. When BDNF mRNA content in blood was normalised over EAR, we found no differences among control (n = 6), preHD (n = 10), and HD subjects (n = 21) (P>0.05 by Kruskal-Wallis test followed by Dunn's multiple comparison test) (**Fig. 3C**). Similar results were obtained on two additional sets of samples (16 control, 11 preHD, and 24 HD samples) from UK Cohort C (P>0.05 by Kruskal-Wallis test followed by Dunn's multiple comparison test) (**Fig. 3D**), and 23 control, 4 preHD, and 24 HD samples from Italian Cohort C (**Fig. 3E**) (P>0.05 by Kruskal-Wallis test followed by Dunn's multiple comparison test). These analyses confirmed that the blood level of BDNF mRNA is similar in control and HD patients.

### Validation of previously identified transcriptomic biomarkers

**Figure 4:**
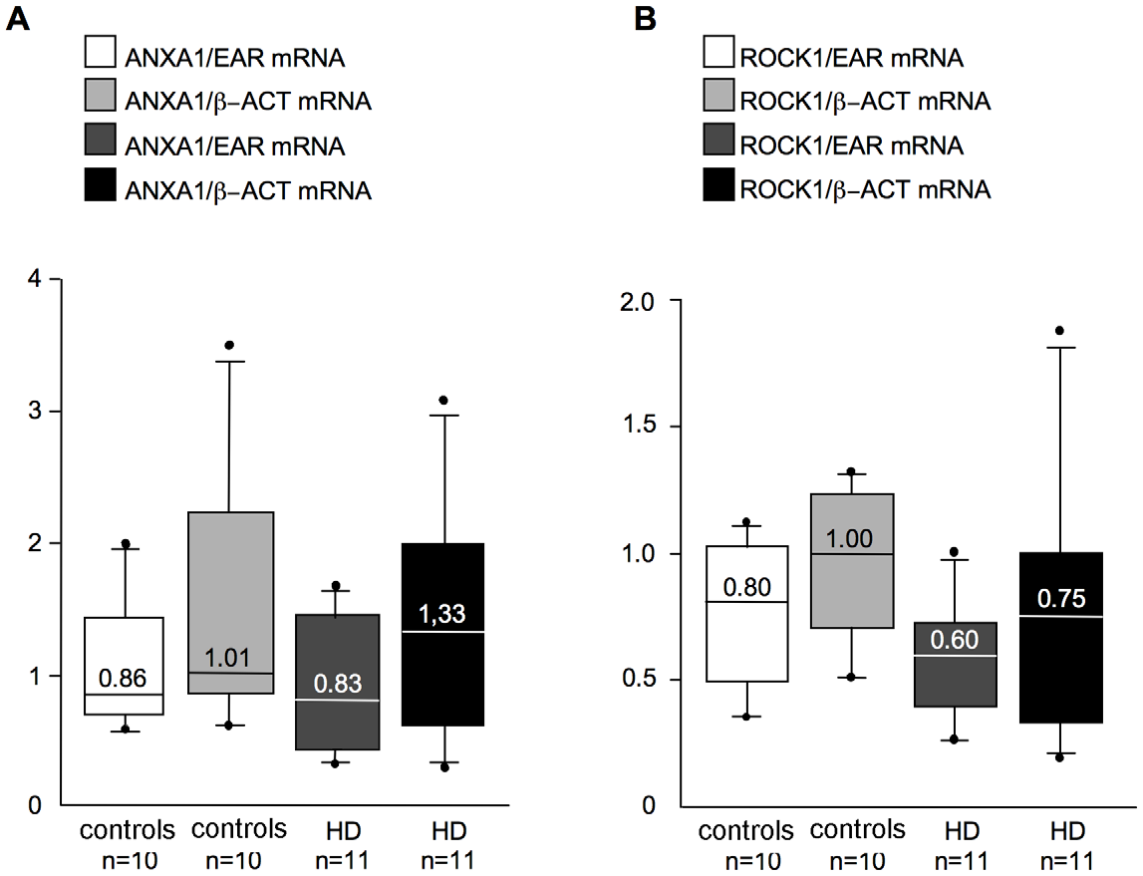
mRNA levels of other HD-related genes in control and HD patients. Samples are from Italian Cohort C. Blood mRNA levels of (**A**) ANXA1 and (**B**) ROCK1 were measured with two different normalisation strategies (ß-actin mRNA levels (ß-ACT), according to [25], and EAR levels, according to [24].

A previous microarray study identified a large number of significantly altered mRNAs in HD blood, from which 12 genes were selected to discriminate between controls and HD patients [25]. Some of the HD-related changes in the expression of these 12 genes were confirmed in one independent study [26], but not in a second one [27]. Here we analysed the expression of 2 of the putative marker genes in a selection of samples from the Italian Cohort C that were previously tested for BDNF mRNA content. We focused on the annexin A1 (ANXA1) and rho-associated protein kinase-1 (ROCK1) genes, because their expression was upregulated in HD blood and was sensitive to pharmacological treatment [25], [26]. Our RT-qPCR experiments indicated that ANXA1 and ROCK1 mRNA levels were similar in control and HD patients (**Fig. 4A–B**). Two different normalization factors were used, ß-actin, as reported in the original article [25] (ANXA1: P>0.05 controls vs HD; ROCK1: P>0.05 controls vs HD, Mann-Whitney test) and EAR [24] (ANXA1: P>0.05 controls vs HD; ROCK1: P>0.05 controls vs HD, Mann-Whitney test), with similar results although normalization over ß-actin caused increased variability. Our results were consistent with previous findings from Runne et al., [27], who used a larger number of cases and the same qPCR primer pairs for the selected targets, but also saw no differential expression in ANXA1 and ROCK1 between preHD and control samples. Our data confirm that transcriptomic analyses of these individual genes may not be suited for tracking HD onset and progression in human blood and that a reliable normalisation procedure, such as EAR, is necessary.

## Discussion

This multicentre study included the largest number of HD subjects, to date, that have been tested for peripheral BDNF mRNA and protein expression, and represents the most comprehensive experimental analysis of BDNF in peripheral human blood. We show that measuring BDNF in the blood of patients with HD was not informative of disease onset or progression. Moreover, we have demonstrated that many issues concerning BDNF protein detection in blood (in particular in serum) may make reliable measurements difficult. This is true for HD, but could be extended to other diseases and to all assays in which serum and plasma BDNF levels have been measured. We also showed that, with the appropriate normalisation procedures [24], the evaluation of BDNF mRNA revealed no differences between control and HD subjects. In spite of this, we propose pPCR against EAR [24] as a precise and reliable strategy to detect the level of BDNF mRNA in total blood as well as the level of other mRNAs.

### The measure of circulating BDNF protein

In this study, we analyzed samples from a total of 91 controls, 31 preHD, and 134 HD subjects. We found that the detection of BDNF protein in human blood samples (plasma and, in particular, serum) is extremely complex. In particular, variability in serum is still prohibitive for consideration as a routine clinical tool, and caution should be exercised when undertaking research studies of BDNF in such a compartment or in plasma from pre-existing banks. First, in our study, different results were obtained when BDNF plasma levels were measured in different cohorts. Second, intra-group variability can be a major problem especially since BDNF levels are affected by a number of individual factors [22]. Third, several methodological aspects related to blood collection, sample storage and BDNF protein detection may contribute to the variability of its measurement in blood. While considering this, no differences in BDNF protein content were found between control and HD patients (in both plasma and serum).

#### Critical aspects to consider when approaching BDNF protein detection in plasma samples

We found that sample collection and preparation may influence the level of BDNF protein measured in plasma. Particularly, we found that the time spent on the bench at RT before plasma preparation affects BDNF content in plasma (**Fig. 1A**). Thus, more robust and standardised procedures are required for plasma preparation. BDNF was enough stable when plasma was stored at −80°C, even after 1 year (data not shown). This is due to the presence of EDTA in blood collection tubes, which reduces the activity of proteases, thus increasing BDNF protein stability. Although proteases are not completely inactivated in plasma samples, our studies and previous studies demonstrate that plasma is intrinsically more stable than serum and therefore, more useful for protein analyses in biomarker studies [28]. Plasma banks might be a suitable method of storing plasma samples for future BDNF protein analyses. However, if the disease of interest is a rare genetic disease and only small cohorts of patients are available, small variations in the protocols used for different blood banks may introduce issues related to plasma sample preparation that affect BDNF measure. In fact, we show here that plasma samples of control and HD subjects from two independent Cohorts showed different BDNF levels (**Fig. 1B** and **Fig. 1C**). The effort conducted within the EURO-HD network (http://www.euro-hd.net/html/network) in coordinating Clinical Centres within Europe is a very welcome and worthwhile strategy as it aims at the application of standardised protocols in response to criteria identified by specialists of tissue banking and also encourages sample deposition in a single bank (for HD studies: BioRep s.r.l. in Milano, http://www.biorep.com). This effort is sustained also by the fact that BDNF ELISA works well on plasma samples (**Text S1**).

#### Testing BDNF protein in serum samples: issues that remain to be resolved

Compared to our findings on BDNF in plasma, we noticed that BDNF appears to be less stable in serum samples prepared following standardised protocols (see **Material and Methods**) and stored at −80°C. The aspect of protein instability is serum is well known to enzymologists and biochemists [29], [30]. Proteolytic enzymes in the clotting cascade may greatly, and randomly, affect the level of a given protein in serum samples. The activity of proteolytic enzymes may continue also with serum storage, causing sample variability and instability by sequential protein degradation. We have found that BDNF is unstable in frozen samples stored at −80°C as previously raised by other authors who showed that serum BDNF is rapidly degraded with storage at −20°C [20]. Moreover, the collection of blood in tubes containing protease inhibitors has little effect on serum protein preservation [31]. Instability of BDNF protein in serum samples clearly emerges from the high inter-assay variation observed in our study also when the same samples have been tested after one week. This is not due to the ELISA assay itself that has been extensively validated in cells, brain tissue and plasma samples [22], [23], [32]. Based on these considerations, one could think to analyse serum samples immediately after preparation. However, if the disease under investigation is rare, it will be very difficult to recruit a number of patients large enough for statistical analyses. In addition, we found that inter-assay variation still remains high when the same serum (but not plasma) samples were analysed in parallel, on the same day, with the same BDNF ELISA kit and solutions, by two different operators (JS and BV) (**Fig. 2D**). This result is difficult to interpret. The only possible explanation at this stage is that serum samples may be subjected to uncontrolled biological (and technical) variations. We suggest that protein stability in serum samples still remain a complicated and unsolved issue that renders serum samples inappropriate for the detection of BDNF (and likely also other proteins) in biomarker studies.

#### Data about BDNF in blood need to be critically re-interpreted

Our study suggests also that changes in serum and plasma samples previously reported to be associated with several disorders might be considered with cautions. By using the PubMED search engine with the keywords “BDNF and human and serum” and “BDNF and human and plasma” we analysed the literature published between 2003 and May 1, 2011 on BDNF in blood. 351 articles emerged from the first search while 288 for the second, some of which could be overlapping. We have selected 30 papers reporting BDNF measurement in human serum and 20 papers in human plasma that contained enough methodological aspects for a critical analysis (**Table S1** and **Table S2**). Even among these cases there are reports in which BDNF protein concentration were described with limited details. BDNF measured in serum and plasma was variable under different pathological conditions, but also among control groups. Serum BDNF content varied from approximately 0.143 to 80 ng/ml, and plasma BDNF content varied from 10 to 10000 pg/ml. Such a high variability can be due to different criteria for subject selection, sample collections and methodological aspects that can seriously affect BDNF detection in blood. Criteria for patient selection are not always available in published papers. In addition, most studies have not indicated whether measurements were made under fasting or fed conditions, as it is also known that diet can influence BDNF levels [22]. Most papers do not sufficiently describe the way blood was collected. Lommatzsch et al., (2005) have shown that different types of blood collection tubes (EDTA or heparin-treated) can influence the BDNF content detected in plasma [17]. Many papers do not contain information on the time and conditions of blood storage that strongly influence the amount of BDNF detected in plasma (see **Fig. 1A**). Temperatures and time of storage of plasma and serum samples vary among studies, thus affecting the absolute amount of BDNF detected. We and others have found that this is true especially for serum samples [18], [20]. Different ELISA assay kits have been used with different range of sensitivity, thus contributing to introduce variability. However, the two most used ELISA kits (from Promega and R&D Systems) efficiently detect BDNF. Underestimated methodological aspects can affect data interpretation and make it difficult to reach any strong conclusion about blood BDNF protein content in different pathological conditions.

### The HD mutation did not affect BDNF mRNA in human peripheral blood

A large number of *in vitro* and *in vivo* studies have shown that BDNF mRNA is consistently reduced in brain tissues from HD transgenic mice and patients [10], [22]. In addition, blood BDNF mRNA levels correlated with brain BDNF levels and disease progression in HD mice [11], [12]. Importantly, a standardised, accurate and fast assay is available to quantitatively evaluate BDNF mRNA in human blood samples [24]. These studies initiated our investigation of the levels of BDNF mRNA in blood.

Our analyses of samples from 47 control, 25 preHD, and 70 HD subjects indicated that, in contrast to findings in HD mouse models, BDNF gene transcription was not affected in the peripheral blood of HD patients. This apparent absence of BDNF modulation in blood may arise because the transcriptional regulatory mechanisms are different in human blood and human brain. The human BDNF gene is characterised by very complex regulation as it contains nine different promoters that act independently to modulate the transcription of BDNF mRNA in a stimulus- and time- specific manner [33]. We previously demonstrated that transcription from the BDNF promoters II, IV, and VI was reduced in the brains of HD animal models and in human postmortem brain tissue [10]. In human blood, most of the BDNF is produced from mRNA isoform IX, with some very moderate involvement of mRNA I and IV (the latter being detectable only at very high PCR amplification cycles; **Figure S1**) while others, including those implicated in brain and affected by the HD mutation appeared to be silent (such as mRNA isoform II; **Figure S1**). These differences in BDNF isoforms expression between blood and brain may account for the differences observed in the effects of the HD mutation on BDNF gene transcription in these two compartments.

Furthermore, microarray studies on blood samples from healthy subjects have shown intra-individual variation in the expression of several genes due to differences in the relative proportions of specific blood cell subsets, gender, age, and the time of day at which the blood was sampled [34]. These factors may also influence the levels of BDNF mRNA in blood. Consistently, it was not surprising that the levels of previously identified serum mRNA markers (ROCK1 and ANXA1) were unaffected by HD in our cohort of samples and in previous studies [25], [27].

### Conclusion

Although BDNF loss in the HD brain is implicated in the disease process [10], this study indicates that the level of BDNF in human blood is not informative (mRNA levels and plasma protein level) or reliable (protein levels in serum) as a biomarker for predicting HD onset or progression.

## Supporting Information

Figure S1 **A**) Correlation between BDNF levels in preHD subjects and time to disease onset. **B**) and **C**) Semiquantitative RT-PCR analyses of human BDNF mRNA isoforms expression in adult human tissues. cDNA samples were amplified for (B) 35 and (C) 40 cycles. In blood (bl), transcripts were predominately detected from promoter IX, and at very low levels, from promoters I and IV. B, blank or RT-.
(DOC)

Text S1 **BDNF ELISA interassay variability.**
(DOC)

Table S1 **Selection of papers about BDNF measure in serum in different pathological conditions.**
(XLS)

Table S2 **Selection of papers about BDNF measure in plasma in different pathological conditions.**
(XLS)
