# Supplementary material for: Brain-Derived Neurotrophic Factor in Patients with Huntington's Disease
Source: PLoS One. 2011 Aug 12;6(8):e22966. doi: 10.1371/journal.pone.0022966 (PMC3155522; doi:10.1371/journal.pone.0022966)
Supplement: Figure S1 — A) Correlation between BDNF levels in preHD subjects and time to disease onset. B) and C) Semiquantitative RT-PCR analyses of human BDNF mRNA isoforms expression in adult human tissues. cDNA samples were amplified for (B) 35 and (C) 40 cycles. In blood (bl), transcripts were predominately detected from promoter IX, and at very low levels, from promoters I and IV. B, blank or RT-. (DOC) [file pone.0022966.s001.doc]

**Figure S1. A**) Correlation between BDNF levels in preHD subjects from UK Cohort A and time to disease onset. **B**) and **C**) RT-PCR analyses of human BDNF mRNA isoforms expression in adult human tissues. cDNA samples were amplified for (**B**) 35 and (**C**) 40 cycles. In blood (bl), transcripts were predominately detected from promoter IX, and at very low levels, from promoters I and IV. ctx, cortex; B, blank or RT-.
