# Supplementary material for: Brain-Derived Neurotrophic Factor in Patients with Huntington's Disease
Source: PLoS One. 2011 Aug 12;6(8):e22966. doi: 10.1371/journal.pone.0022966 (PMC3155522; doi:10.1371/journal.pone.0022966)
Supplement: Text S1 — BDNF ELISA interassay variability. (DOC) [file pone.0022966.s002.doc]

**BDNF ELISA interassay variability**

**Plasma**: operator Alessia Tarditi, AT. The analyses have been performed on aliquots of plasma that have never been de-freezed before. ELISA 1, 2 and 3 have been performed respectively on 20-11-2006; 21-11-2006, 22-11-2006. Sample dilutions (1:20) have been performed the same day of the assay. Intra-assay CVs were <11%.

|  | *ELISA n.1 pg/ml* | *ELISA n.2 pg/ml* | *ELISA n.3 pg/ml* | *Average pg/ml* | *SD* | *CV(%)* |
| --- | --- | --- | --- | --- | --- | --- |
| Plasma sample # A | 172 | 144 | 149 | 155 | 15 | 9,6 |
| Plasma sample # B | 639 | 560 | 689 | 629 | 66 | 10,3 |
| Plasma sample # C | 1801 | 1451 | 1614 | 1622 | 175 | 10,8 |

**Serum**: operator Barbara Vitali, BV. The analyses have been performed on aliquots of serum that have never been de-freezed before. ELISA 1, 2 and 3 have been performed respectively on 8-10-2010, 15-10-2010, 20-01-2010. Sample dilutions (1:300) have been performed the same day of the assay. Intra-assay CVs were very high with values between 38,1 and 99,4%.

|  | *ELISA n.1 pg/ml* | *ELISA n.2 pg/ml* | *ELISA n. 3 pg/ml* | *Average pg/ml* | *SD* | *CV(%)* |
| --- | --- | --- | --- | --- | --- | --- |
| Serum sample # A | 6807 | 1212 | 2052 | 3357 | 3017 | 89,9 |
| Serum sample # B | 12325 | 12072 | 5368 | 9921 | 3945 | 39,8 |
| Serum sample # C | 8486 | 1652 | 2052 | 4063 | 3835 | 94,4 |
| Serum sample # D | 6584 | 2983 | 158 | 3241 | 3220 | 99,4 |
| Serum sample # E | 14705 | 5212 | 2810 | 7576 | 6289 | 83,0 |
| Serum sample # F | 10097 | 8442 | 4429 | 7656 | 2914 | 38,1 |

As described in the paper, to try to limit inter-assay variations in the BDNF analyses on serum we have organized our assay in order to thaw all serum samples on the same day and BV and Jenny Sassone (JS) have worked in parallel on the same day and with the same samples, ELISA assays, reagents, solutions, and bench. Intra-assay CVs were very high with values between 27,0 and 72,8%.

|  | *Operator BV pg/ml* | *Operator JS pg/ml* | *Average pg/ml* | *SD* | *CV(%)* |
| --- | --- | --- | --- | --- | --- |
| Serum sample # A | 5368 | 11034 | 8201 | 4006 | 48,9 |
| Serum sample # B | 10192 | 15013 | 12603 | 3409 | 27,0 |
| Serum sample # C | 2052 | 6404 | 4228 | 3077 | 72,8 |
| Serum sample # D | 2810 | 6813 | 4811 | 2830 | 58,8 |
| Serum sample # E | 4428 | 7047 | 5738 | 1852 | 32,3 |
